# Genotyping results of Iranian PCG families suggests one or more PCG locus other than GCL3A, GCL3B, and GCL3C exist

**Published:** 2009-10-22

**Authors:** Mehrnaz Narooie-Nejad, Fereshteh Chitsazian, Betsabeh Khoramian Tusi, Faride Mousavi, Massoud Houshmand, Mohammad R. Rohani, Azam S. Hosseinipour, Akram Rismanchian, Elahe Elahi

**Affiliations:** 1 National Institute of Genetic Engineering and Biotechnology, Tehran, Iran; 2 Department of Cellular and Molecular Biology, School of Biology, College of Science, University of Tehran, Tehran, Iran; 3 Tabriz University of Medical Sciences, Nikoo-kari Hospital, Tabriz, Iran; 4 Zahedan University of Medical Sciences, Zahedan, Iran; 5 Division of Specialized Education, Ministry of Education, Tehran, Iran; 6 Esfahan University of Medical Sciences, Esfahan, Iran; 7 Center of Excellence in Biomathematics, Statistics, and Computer Science, College of Science, University of Tehran, Tehran, Iran.

## Abstract

**Purpose:**

To assess whether loci other than GLC3A, GLC3B, and GLC3C are linked to primary congenital glaucoma (PCG).

**Methods:**

The gene *CYP1B1* at GLC3A was screened in 19 Iranian PCG probands who had been recruited mostly from among individuals of Turkish ethnicity and individuals from central and eastern Iran. The gene *MYOC* was screened in patients from this cohort who lacked *CYP1B1* mutations and in ten patients previously shown not to carry *CYP1B1* mutations. Family members of 19 probands without mutations in either of these genes were recruited for assessment of linkage to GLC3B and GLC3C by genotyping microsatellite markers. In total, 127 individuals, including 35 affected with PCG, were genotyped.

**Results:**

Eleven (57.9%) of the newly recruited PCG patients did not carry disease-associated mutations in *CYP1B1*. Disease-associated *MYOC* mutations were not observed in any of the patients screened. Inheritance of PCG in all the families was consistent with an autosomal recessive pattern. Linkage to GLC3B and GLC3C was ruled out in nine of the families on the basis of autozygosity mapping and haplotype analysis.

**Conclusions:**

Observation of the absence of linkage to GLC3B and GLC3C in at least nine families without *CYP1B1* mutations suggests that at least one PCG-causing locus other than GLC3A, GLC3B, and GLC3C may exist. The disease-causing gene or genes in the novel locus or loci may account for PCG in a notable fraction of Iranian patients.

## Introduction

Primary congenital glaucoma (PCG; OMIM 231300) is a severe form of glaucoma that develops early in life, usually in the neonatal period or before the age of three years [1]. It is characterized by an anatomical defect in the trabecular meshwork (trabeculodysgenesis), and its clinical features include increased intraocular pressure, globe enlargement (buphthalmos), corneal enlargement, Descemet’s membrane rupture, corneal edema and opacification, and optic nerve damage. PCG occurs in both familial and sporadic patterns [1,2]. Inheritance in familial cases is usually autosomal recessive. The incidence of PCG is geographically and ethnically variable, estimated at 1:10,000 in Western countries and higher in inbred populations — for example, 1:2,500 in Saudi Arabia [1,2].

Three PCG loci have been identified by linkage analysis in multiply affected families: GLC3A [3], GLC3B [4], and GLC3C [1,5] (GLC is the designation used for glaucoma loci, and the numeral 3 refers to the congenital form). The gene associated with GLC3A, cytochrome P450, family 1, subfamily B, polypeptide 1 (*CYP1B1*; OMIM 601771), was identified in 1997 [6]. *CYP1B1* is a member of the cytochrome P450 superfamily of genes. Although physiological studies have confirmed that mutations in *CYP1B1* can cause disease, the pathway by which *CYP1B1* affects development of the anterior chamber of the eye is unknown. Presumably, mutations in the gene result in aberrant metabolism of a PCG-relevant endogenous substrate of the coded cytochrome P450 enzyme. The proportion of PCG patients whose disease is due to *CYP1B1* mutations is generally high, but it varies among populations, ranging from 100% in Slovakian Roma to ~20% in Japan [7,8]. It has been observed that nearly 70% of an Iranian cohort of PCG patients carried disease-associated mutations in *CYP1B1* and that 30% did not [9]. Although more than ten years have passed since the identification of the GLC3B locus, the disease-causing gene at this locus has not yet been identified [4]. GLC3C was reported in 2002. Recently, PCG-causing mutations in latent transforming growth factor beta binding protein 2 (*LTBP2*; OMIM 602091) were identified in Pakistani, European Gypsy, and Iranian patients [10,11]. *LTBP2* lies very close to GLC3C on chromosome 14, but it is not strictly within the locus as originally defined by microsatellite markers [5]. As such, it is not clear whether *LTBP2* is the PCG-associated gene within GLC3C or whether the gene within this locus remains unknown and *LTBP2* defines a fourth locus for PCG. Based on structural properties, LTBP2 is a member of a superfamily of proteins composed of fibrillins and latent transforming growth factor beta binding proteins [12–14]. Although the precise function of LTBP2 remains unknown, there is evidence for LTBP2 having roles in tissue repair processes, cell adhesion, and functions related to those of microfibrils and elastin fibers [15–17]. In addition to said loci and genes associated with PCG, mutations in the gene coding myocilin (*MYOC*) — a gene generally associated with early-onset primary open angle glaucoma — occasionally have been reported in PCG patients [18,19].

In this report, we show by means of local autozygosity mapping and haplotype analysis restricted to chromosomal regions of known loci in 19 Iranian PCG families without *CYP1B1* and *MYOC* mutations that at least one novel PCG-associated locus is likely to exist. The assumption behind autozygosity mapping is that an affected offspring born to consanguineous parents has inherited disease-causing alleles that are identical by descent from both parents. In the case of families with multiple affected offspring, the assumption is that the genetic cause of PCG is the same for all affected siblings within any one family. In such situations, one would expect all the affected children to have inherited the same chromosome from their mother and from their father. This would be reflected by the children harboring the same pair of haplotypes, which are combinations of alleles for markers on those chromosomes. Based on these criteria, disease status in at least 11 of the families did not link to any of the known PCG loci.

## Methods

This research was performed in accordance with the Declaration of Helsinki. All participants or their responsible guardians consented to participate after being informed of the nature of the research. Among the 32 PCG patients previously shown not to harbor *CYP1B1* mutations, 16 cases were familial, and the family members of ten patients were available for further study by microsatellite genotype analysis [9]. Another 19 PCG probands were recruited from Nikoo-kari (Tabriz, Iran), Farabi (Esfahan, Iran), and Al-Zahra (Zahedan, Iran) hospitals and from schools for blind children located in the same cities as the hospitals. The clinical features of the newly recruited patients were obtained from hospital and school records. All patients had been examined at some point previous to this study by one or more glaucoma specialists. Four of these patients were blind in one or both eyes at the time of recruitment. Recorded information on two patients was considered insufficient, and these were reexamined by a glaucoma specialist at Al-Zahra hospital in Zahedan. Criteria for PCG diagnosis included intraocular pressure ≥21 mmHg in at least one eye; corneal edema; Descemet’s membrane rupture; megalocornea (corneal diameter ≥12 mm); and high cup-to-disc ratio, suggesting glaucomatous optic nerve head damage. Patients with other ocular or systemic anomalies were excluded. The newly recruited patients were screened for mutations in *CYP1B1* by direct sequencing [9]. All patients from the previous and new cohorts without disease-associated mutations in *CYP1B1* were screened by direct sequencing for mutations in *MYOC* [20]. Sequencing of the *CYP1B1*and *MYOC* genes were done using the ABI BigDye terminator chemistry and an ABI Prism 3700 instrument (Applied Biosystems, Foster City, CA). Family members of patients without *CYP1B1* and *MYOC* mutations were then recruited for local autozygosity mapping and haplotype analysis of loci GLC3B and GLC3C.

Ultimately, microsatellite genotyping analysis was done on 19 families. A total of 127 individuals, including 35 PCG-affected individuals, belonging to the 19 families were genotyped. All individuals designated unaffected were ten years old or older at time of recruitment. GLC3B spans 1.04 Mb on chromosome 1, and four polymorphic microsatellites were used to assess absence of linkage to this locus: tel-D1S228 - D1S402 - D1S407 - D1S507-cen tel-D1S228 D1S402 D1S407 D1S507-cen [4]. GLC3C spans 5.77 Mb on chromosome 14, and five polymorphic microsatellites were used to assess absence of linkage to this locus: cen-D14S61 - D14S263 - D14S1020 - G10271 - D14S1000-tel cen-D14S61 - D14S263 - D14S1020 - G10271 - D14S1000-tel [5]. These markers span close-flanking regions as well as the loci themselves. The sequences of primers used for PCR amplification of DNA segments containing these microsatellites are provided in Table 1. The forward primers were fluorescently labeled with 6- carboxyfluorescein  (6-FAM), 2'-chloro-5'-fluoro-7',8'-fused phenyl-1,4-dichloro-6-carboxyfluorescein (NED), or hexachloro-*fluorescein*(HEX) in combinations that allowed maximal multiplexing. The size of the amplicons was determined using ABI3730XL (Applied Biosystems, Foster City, CA). Assessment of absence of linkage was established by visual inspection. Absence of linkage to GLC3B or GLC3C was surmised if affected children born to consanguineous parents were found not to be homozygous for the respective markers. In cases of affected siblings born to non-consanguineous parents, absence of linkage was surmised if the affected children did not share common haplotypes for the respective markers, irrespective of homozygosity. For apparently sporadic cases, linkage was considered possible if homozygosity was observed, and no assessment concerning linkage was made if heterozygosity was observed.

**Table 1:** Sequences of primers used for amplification of microsatellite markers.

**Marker name**	**Forward primer**	**Reverse primer**
GlC3C Markers		
D14S61	5'-GTTCCTGCTAAAAGTCAAGTGG-3'	5'- TCAGAGAGGAAGGTTGGACTG -3'
D14S263	5'-TCATCACAGGCCTTCCTATCC-3'	5'-CTAGGACTTGGCGAATGGTTG-3'
D14S1020	5'-GCCTTTACAGAGGGACTCATC-3'	5'-TCTACTGGGAGCTAGGGCAC-3'
G10271	5'-TCTTAGCCAAAAAGTAGACAGTG-3'	5'-AGGACAGGCTACACCCACAG-3'
D14S1000	5'-TTGTATTGCCAACTGGTTGGTG-3'	5'-CCTACTTATGCTTGGGTACACA-3'
GLC3B Markers		
D1S228	5'-AAATAACTGCAACATTGAAATGGC-3'	5'-GGGACCATAGTTCTTGGTGAC-3'
D1S402	5'-TAGACAATAGAGTGAGATTTCAG-3'	5'-TATGGCACTTGGAAATTGACTG-3'
D1S407	5'-CTGTGCTAACCACATGGAGAG-3'	5'-AGCACTTCATTCACTTGTCTGG-3'
D1S507	5'-GAGGGGATCTTGGCACTTGG-3'	5'-CTAGGGTTTCTGGAAAATGCTG-3'

## Results

**Figure 1:**
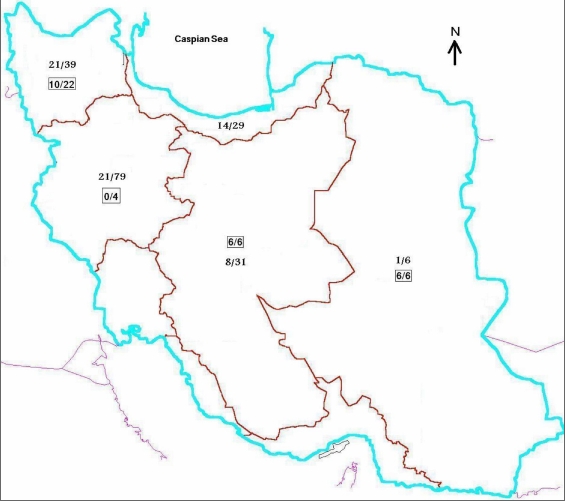
Distribution in Iran of primary congenital glaucoma patients who do not carry *CYP1B1* mutations. The number of chromosomes of primary congenital glaucoma (PCG) patients recruited from a given area of Iran that were observed not to carry *CYP1B1* mutations is shown as the numerator of each fraction, and the total number of chromosomes screened in the areas is shown as the denominator. Fractions not enclosed in boxes relate to an earlier screening effort [9], and fractions enclosed in boxes refer to the more recent recruitment, which focused on northwestern, central, and eastern Iran.

The 104 Iranian PCG-affected individuals previously screened for mutations in *CYP1B1 *consisted mostly of patients recruited from hospitals in Tehran that are national reference centers [9]. The ethnicity of 92 of the 104 patients was recorded, and most were from western Iran, consistent with “common knowledge” among glaucoma specialists in Iran that PCG is most prevalent in that part of the country (personal communication, Shahin Yazdani and Naveed Nilforushan). Upon screening for mutations in *CYP1B1*, we observed that 14 of the 26 patients (53.8%) of Turkish ethnicity, mostly from northwestern Iran, did not harbor mutations in *CYP1B1*, whereas only 16 of the 66 remaining patients with known ethnicity (24.2%) lacked *CYP1B1* mutations (Figure 1) [9]. As one objective of our research on glaucoma was to identify unknown loci and genes associated with PCG, our further patient recruitment efforts focused in part on the northwestern region of Iran and on individuals of self-reported Turkish ethnicity. Additionally, we emphasized central and eastern Iran, regions of the country that were notably underrepresented in the PCG cohort recruited from the national glaucoma reference centers in Tehran. This approach proved to be fruitful, as 11 of the 19 newly recruited PCG probands (57.9%) were observed not to harbor disease-associated mutations in *CYP1B1*, compared with 32 of 104 patients (30.8%) previously recruited at national reference centers in Tehran (Figure 1). The *CYP1B1* mutations present in eight of the new probands were E37X, G61E (two patients), E229K, R368H, R390H (two patients), and F445S. All eight individuals harbored the mutations in the homozygous state. Mutations in *MYOC* were not observed in any PCG patients without *CYP1B1* mutations among either the previous or the new cohort. Two of the 11 new patients at this point chose not to participate in the microsatellite genotype analysis, leaving 9 newly recruited patients and 10 patients from our previous recruitment to take part in this portion of the study. The clinical features of these 19 probands are presented in Table 2.

**Table 2:** Clinical features of Iranian PCG patients without *CYP1B1* and *MYOC* mutations.

Family number*			**Gender**	**Affected eye**	**Age of onset**	**IOP max (mmHg) R/L**	**Megalo-cornea**	**C/D ratio R/L**	**Corneal opacity (R/L)**	**Edema R/L**	**Haab's striaie (R/L)**	**Familial status**			**Trabecu-lotomy**
												**Sporadic**	**Consang. parents**	**Non-consang. parents> 1 affected**	
**Maybe B^b^**	**Maybe C^c^**	**Not B, Not C^x^**	**Linkage unknown^y^**													
																
CGL-201				M	Unilateral	2 months	10/18.5	+/+	0.8/0.2		+/-	+/+	+			-
	CGL-202			F	Bilateral	3 months		+/+		++/++	+/+			+		+
		CGL-203		M	Bilateral	5 months								+		+
			CGL-205	M	Unilateral	8 months	16/17	-/-	0.2/0.9	-/-	-/-	-/-	+			-
CGL-206				M	Bilateral	at birth	#/18	+/+	#/0.7	#/-	-/-	+/+		+		-
	CGL-207			F	Bilateral	4 months	34/24	-/+	0.3/0.2	+/+	-/-	-/+		+		+
CGL-209				F	Bilateral	5 months	22/22	+/+	0.3/0.8	-/-	-/-	-/-		+		+
			CGL-210	M	Bilateral	2 months	13/12	-/-	0.2/0.2	-/-	-/-	+/+	+			-
	CGL-211			M	Unilateral	at birth	30/14	-/+	0.3/0.6	+/+	-/-	-/-	+			+
		CGL-212		F	Bilateral	at birth	22/27							+		+
	CGL-217			M	Bilateral	at birth	EN/24	EN/+	EN/0.95	EN/++	EN/+	EN/-		+		+
		CGL-218		M	Bilateral	3 years	24/EN		0.9/EN	+/++	-/++	+/+		+		-
		CGL-221		F	Bilateral	at birth	27/#	l-/#	0.8/#	l-/#	-/+				+	+
		CGL-222		M	Bilateral	4 months	32.5/26							+		-
		CGL-223		F	Bilateral	5 months	24/13								+	+
		CGL-224		F	Bilateral	4 months	19/22.5	+/+	0.2/0.2	+/+	-/-	+/+		+		+
	CGL-225			F	Bilateral	6 months	24/17	+/+	0.8/0.6	-/-	+/+			+		+
		CGL-226		F	Bilateral	at birth	EN/15	EN/+	EN/0.9	-/-				+		+
		CGL- 227		M	Unilateral	3 months	28/18	+/+	0.8/0.9	+/+	-/-	+/+		+		+

Unknown phenotypic features are left blank. * Features of probands are presented. b: Linkage to GLC3B not rejected in these families ; c: Linkage to GLC3C not rejected in these families; x: Linkage to GLC3B and GLC3C rejected in these families: y: unable to assess linkage to GLCB and GLCC. #Unable to measure. Abbreviations: C/D ratio Cup/disk ratio of optic nerve; EN: enucleated; R: right eye; L: left eye; M: Male, F: Female.

Affected individuals in 13 of the 19 families genotyped with microsatellite markers were born to consanguineous parents (Table 2). There were multiple affected individuals in two of the six non-consanguineous families, suggesting that PCG in these was also familial. The four remaining families had only one affected child, and the parents reported non-consanguinity, but they were included in the study because the parents in all four cases belonged to highly inbred small villages. PCG appeared in all the families in a manner consistent with autosomal recessive inheritance. Disease inheritance was considered consistent with an autosomal recessive pattern even in the single family (CGL-221) in which affected individuals were observed in two consecutive generations because in this family, the parents themselves had been born to parents who were first cousins.

**Figure 2:**
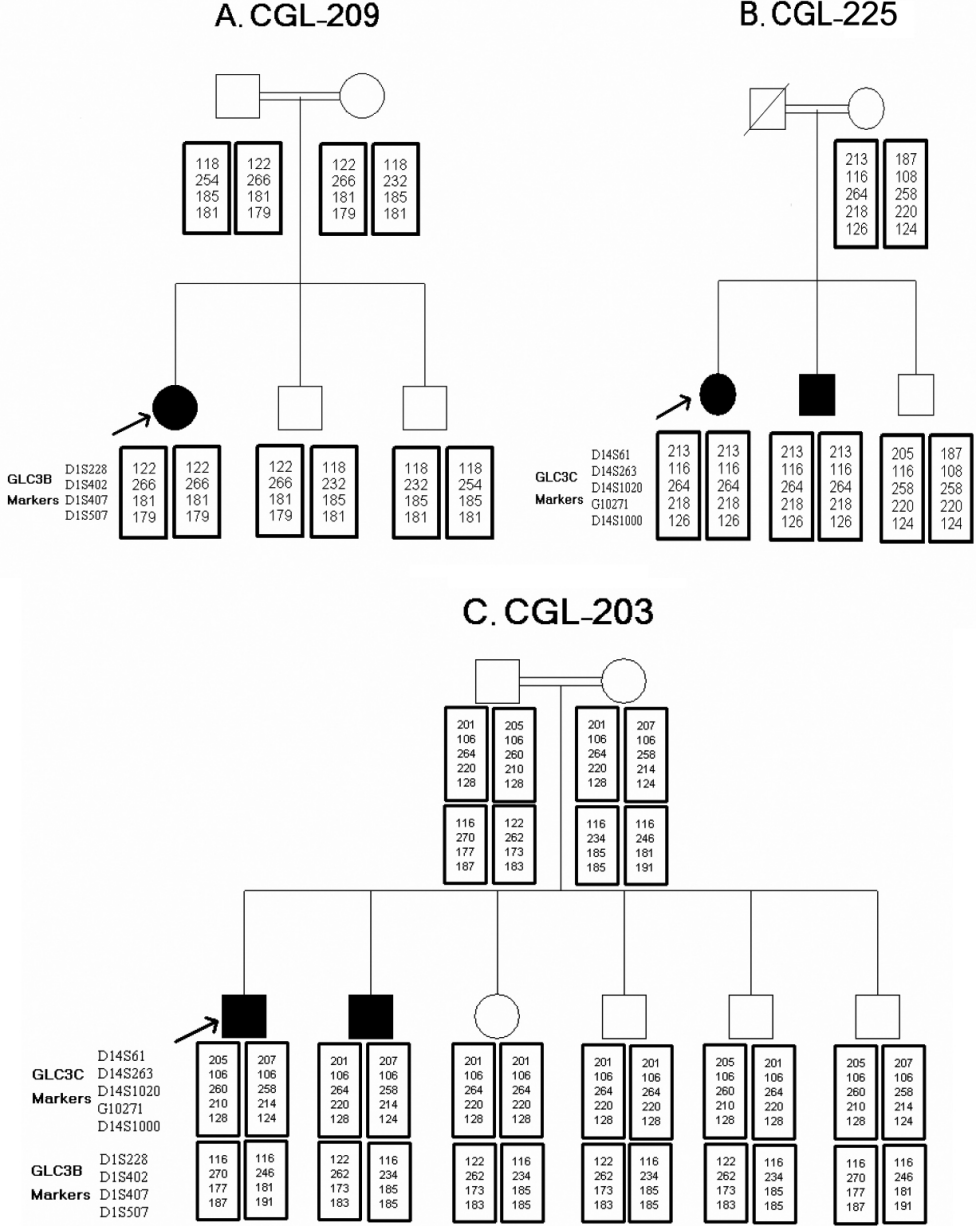
Genotyping results of primary congenital glaucoma families. Sizes (bp) of PCR products of microsatellite markers of genotyped individuals are shown. **A**: Linkage to GLC3B was not rejected in family CGL-209. **B**: Linkage to GLC3C was not rejected in family CGL-225. **C**: Family CGL-203 does not show linkage to GCL3B or to CGL3C.

Linkage of PCG status to GLC3B could not be rejected in one consanguineous family with one affected child because of homozygosity at all markers tested for this locus (Figure 2A). Furthermore, linkage to this locus was considered possible in two other families with one affected individual each, because of homozygosity at one distal marker. Linkage of PCG to GLC3C also could not be rejected in three multicase consanguineous families, as the affected individuals in these families were homozygous for all five markers tested and none of the unaffected individuals were homozygous for all the markers (Figure 2B). Linkage of disease to GLC3C in two additional families also was considered possible, as homozygosity at bordering markers was observed in the affected individuals. PCG appeared sporadic in two of the remaining 11 cases; as homozygosity was not observed in these two cases, linkage to the loci could not be assessed.

Linkage to GLC3B and GLC3C was ruled out in nine families (Table 2). At least one affected child was born to consanguineous parents (first cousins in all cases) in seven of these families, yet homozygosity was not observed for the markers of these loci (Figure 2C). The affected siblings in each of two non-consanguineous families did not share common haplotypes as defined by the microsatellite markers.

## Discussion

We had shown that 75.8% of patients of non-Turkish ethnicity in the PCG cohort of our previous study, most of whom were from western Iran, harbored mutations in *CYP1B1* [10].  This observation**most likely reflects the expansion of founder *CYP1B1 *mutations in that region of the country. This proposition is supported by the observation that despite evidence of approximately 30 disease-causing *CYP1B1 *mutations among Iranian PCG patients, four mutations (G61E, R368H, R390H, and R469W) constituted 76% of the mutated *CYP1B1 *alleles identified. It was shown that chromosomes of different individuals harboring the same mutation all shared a common haplotype as defined by intragenic single nucleotide polymorphism (SNP) markers, suggesting a common origin for these chromosomes [10]. The expansion of these mutations appears to have been more limited in regions beyond western Iran, as reflected by their lower representations among study participants from outside the region. Although PCG prevalence is believed to be highest in western Iran, looking outside this region proved to be useful for identifying PCG families whose disease status is not due to mutations in *CYP1B1*.

Among the PCG probands in the present study, most belonged to families that were not sufficiently large to allow assessment of definitive linkage to GLC3B or GLC3C. Among the families in which linkage to GLC3C could not be rejected, genome-wide autozygosity mapping in two families subsequently confirmed linkage and led to the identification of disease-associated mutations in *LTBP2*, attesting to the power of this approach [11]. (CGL225 is a branch of pedigree A in reference [11], and CGL202 is pedigree B in that reference.) *LTBP2* is proximal to but outside of GLC3C as originally defined [5]. *LTBP2* has not yet been screened in the other families in this study for which linkage to GLC3C was not ruled out. In the present study, local autozygosity mapping restricted to chromosomal regions of known loci was used as a tool to reject linkage to known loci rather than to definitively identify linkage to known loci or to find novel loci. For this purpose, even small pedigrees may be informative. Rejection of linkage to known loci of an autosomal recessive disease is relatively easy in families in which affected children are born to consanguineous parents; absence of homozygous genotypes at critical markers suggests absence of linkage. In non-consanguineous families with multiple affected children, absence of shared haplotypes suggests absence of linkage.

Here, linkage to GLC3B and GLC3C was shown to be unlikely in nine of the 19 families genotyped with microsatellite markers. The families previously had been shown not to harbor mutations in *CYP1B1* at GLC3A. The observations suggest that the PCG-causing genes in a notable fraction of Iranian PCG families likely are not positioned within the three known loci, GLC3A, GLC3B, and GLC3C, and that at least one unknown PCG locus is expected to exist. The same locus or loci may account for disease in a fraction of PCG patients in populations wherein *CYP1B1* is not a common cause of the disease [7,21-24]. We now seek to identify the position of the novel PCG locus or loci by genome-wide genotyping. Identification of genes therein is expected to shed light on the etiology of PCG and may help in the development of improved therapeutics.
